# SimpylCellCounter: an automated solution for quantifying cells in brain tissue

**DOI:** 10.1038/s41598-020-68138-4

**Published:** 2020-07-28

**Authors:** Aneesh Bal, Fidel Maureira, Amy A. Arguello

**Affiliations:** 1 Department of Psychology, Behavioral Neuroscience, Michigan State University, Interdisciplinary Science and Technology Building, West Lab Rm 4100, 766 Service Rd., East Lansing, MI 48824 USA; 2 Biological Systems Engineering, Washington State University, Paccar 351, Pullman, WA 99164-6120 USA

**Keywords:** Cellular neuroscience, Image processing

## Abstract

Manual quantification of activated cells can provide valuable information about stimuli-induced changes within brain regions; however, this analysis remains time intensive. Therefore, we created SimpylCellCounter (SCC), an automated method to quantify cells that express cFos protein, an index of neuronal activity, in brain tissue and benchmarked it against two widely-used methods: OpenColonyFormingUnit (OCFU) and ImageJ Edge Detection Macro (IMJM). In Experiment 1, manually-obtained cell counts were compared to those detected via OCFU, IMJM and SCC. The absolute error in counts (manual versus automated method) was calculated and error types were categorized as false positives or negatives. In Experiment 2, performance analytics of OCFU, IMJM and SCC were compared. In Experiment 3, SCC analysis was conducted on images it was not trained on, to assess its general utility. We found SCC to be highly accurate and efficient in quantifying cells with circular morphologies that expressed cFos. Additionally, SCC utilized a new approach to count overlapping cells with a pretrained convolutional neural network classifier. The current study demonstrates that SCC is a novel, automated tool to quantify cells in brain tissue and complements current, open-sourced methods designed to detect cells in vitro.

## Introduction

Immediate early genes (IEGs) are rapidly transcribed and translated upon stimulus exposure, making them useful for post-behavioral, correlated readouts of cellular activity^1^. cFos, a commonly studied IEG, is a proto-oncogene and member of the Fos family of transcription factors. *cfos* mRNA is transcribed within minutes of stimulus exposure and results in the cytoplasmic expression of cFos protein 60–90 min later^2^. Behaviorally-induced increases in the number of cFos-Immunoreactive (cFos-IR) cells often suggest that these activated neuronal ensembles may contribute to specific behaviors. For example, characterization of the brain regions and cell types in which cFos protein is increased has provided insight into the cell populations that contribute to learning and memory, drug addiction, obesity and fear conditioning^3-11^.

To analyze cFos-IR cells, experimenters can choose between manual or automated methods for quantification. Brain slices are typically immunohistochemically stained for cFos protein, resulting in round, light to dark-labeled cells. For manual quantification, cells can be counted in areas within a set microscopic field of view. Alternatively, digital images of cFos-stained tissue can be obtained, imported and thresholded using software such as ImageJ to aid in counting^4,5,12,13^. While analysis with select software improves the reliability of manual quantification, an experimenter must still determine whether to count a dark-stained cell based on smoothness, clarity and size. Additionally, as the number of images increases so does fatigue and the potential for increased errors in counts.

Existing automated methods can be used to quantify cFos-like cells even though they are optimized for cell colony or tumor spheroid analysis. However, these algorithms encounter problems with edge detection, contrast enhancement and denoising in brain tissue analysis^14-18^. Edge detection allows for clean segmentation of cells within colonies but can detect false edges in background staining present with cFos cells which can obscure the cells of interest. While contrast enhancement makes it easier to detect cFos-IR cells, it can result in an overestimation of total cell count due to increased pixel intensity of dimly-stained cells. Lastly, denoising can remove background noise from cFos images, but it can also lead to false negatives in images where cFos-IR cells may be slightly out-of-focus. Therefore, to increase objectivity, reliability and minimize the time required to analyze images, an improved automated method for quantifying cFos-like cells in brain tissue is required.

**Figure 1:**
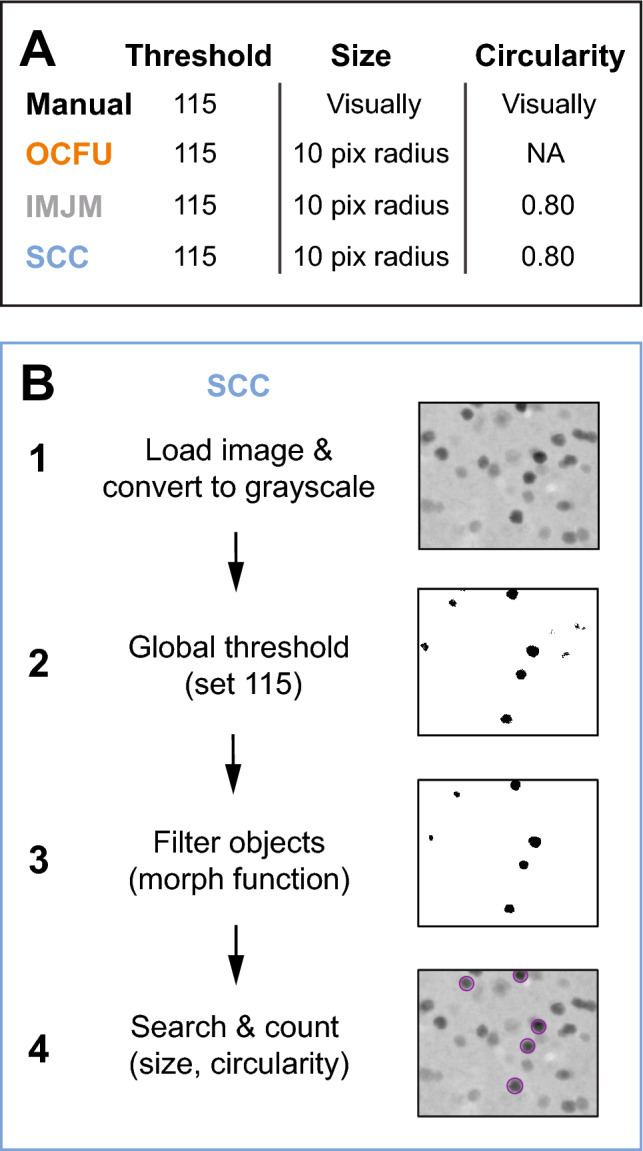
Schematic of automated processing steps. (**A**) Selected parameters for threshold (pixel intensity), object size (pixel radius) and object circularity for Manual, OpenColonyFormingUnit (OCFU), ImageJ Edge Detection Macro (IMJM) and SimpylCellCounter (SCC) of cFos-immunoreactive (cFos-IR) cells. (**B**) Image processing sequence for SCC: (1) loads images and converts to 8-bit grayscale making all pixel intensities range between 0 and 255, (2) performs global threshold based on a user-set value and creates a binarized mask, (3) performs morphological operations on the binary mask to filter out noise-like particles, (4) further selects for cells based on size, circularity and overlap, resulting in a final cell count.

We created SimpylCellCounter (SCC), an efficient and accurate automated method to quantify cFos-IR cells in brain tissue. SCC utilizes binary thresholding and morphological functions from the open-sourced computer vision library OpenComputerVision (OpenCV)^19^, implemented in Python 3. SCC allows a user to manually set parameters of darkness with binary thresholding, cell size and circularity by filtering out non-circular objects and counting only user-defined objects (Fig. 1). SCC utilizes OpenCV’s highly efficient image processing functions to rapidly batch process large sets of digital images and incorporates a new approach to separate overlapping cells via a convolutional neural network.

To test the feasibility and efficiency of SCC, we compared our algorithm to two highly-cited, open-sourced cell colony-based automated quantification methods: OpenColonyFormingUnit^17^ (OCFU) and ImageJ Edge Detection Macro^16^ (IMJM). We chose OCFU and IMJM due to the similarities between cFos-IR cells and the cell colony images analyzed in their respective publications. We used 192 images of cFos-IR cells from the orbitofrontal cortex (OFC) of rats that underwent a cue-induced reinstatement paradigm where previous drug-paired cues elicited increased drug-seeking behaviors^23^. We tested various metrics of performance between OCFU, IMJM and SCC and found that SCC quantified cFos-IR cells with high accuracy when compared with manual analysis. SCC displayed the fastest quantification time of all automated methods tested and maintained accuracy and efficiency when threshold values and image size were adjusted. Lastly, we showed that SCC generalized across multiple sets of images (fabricated cFos images, *S. aureus* and *E. coli* colonies), indicating that it was not overfit to our laboratory’s method of cFos analysis.

## Results

### Comparison of cFos-IR counts between manual and automated methods (Experiment 1)

**Figure 2:**
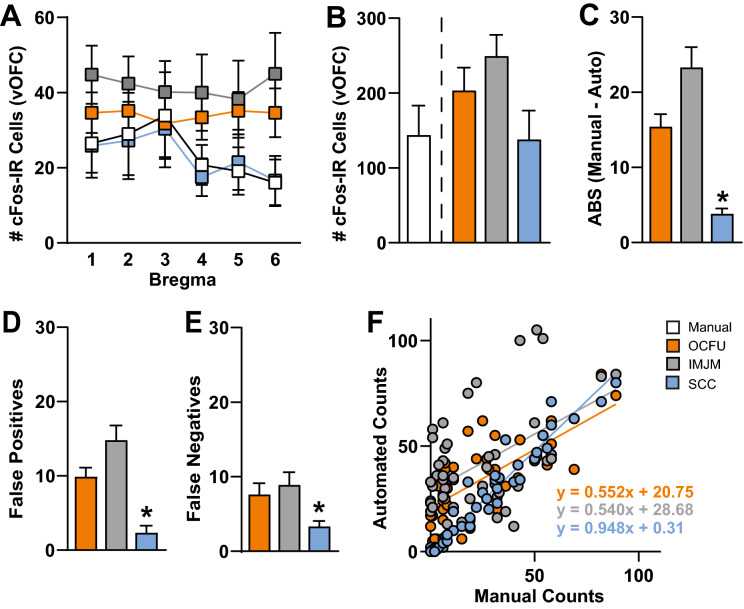
Comparison of Manual versus Automated Quantification Methods. Manual versus automated quantification of cFos-Immunoreactive (IR) cells obtained from the ventral orbitofrontal cortex (vOFC) of rats that underwent cue-induced reinstatement of drug-seeking behavior. The automated methods included: OpenColonyFormingUnit (OCFU, orange), ImageJ Edge Detection Macro (IMJM, gray) and SimpylCellCounter (SCC, blue). (**A**) Average number of cFos-IR cells counted by manual versus automated methods over several points along the anterior–posterior axis (bregma + 5.12 to + 3.72) of the vOFC, n = 96 total images per method. (**B**) Average number cell counts. (**C**) Average absolute error: $ABS\left(Manual\;counts-Automated\;counts\right)$, (**D**) Average number of false positives, number of cells detected by automated methods that were not counted manually, n = subset of 30, (**E**) Average number of false negatives, number of cells counted manually that were not detected by automated methods, n = subset of 30 images, (**F**) Correlation of manual versus automated counts. Manual correlated with OCFU, *p* < 0.001 and regression equation of y = 0.552x + 20.75; Manual correlated with IMJM, *p* < 0.001 and regression equation of y = 0.540x + 28.68; Manual correlated with SCC, *p* < 0.001 and regression equation of y = 0.948x + 0.31.

The number of cFos-IR cells at several bregma points within the ventral OFC (vOFC) was quantified manually (white) or with three automated methods: OCFU (orange), IMJM (gray) and SCC (blue) (Fig. 2). Specifically, a 4 × 6 ANOVA revealed no significant Method x Bregma interaction effect or main effects of Method or Bregma (Fig. 2A). Therefore, all automated methods (OCFU, IMJM and SCC) detected similar average number of cFos-IR cells at each bregma point. An ANOVA of the total number of vOFC cFos-IR cells quantified by each method did not reveal a significant effect. However, there was a trend (F_3, 28_ = 2.51, *p* = 0.079) for an increased total number of cell counts by OCFU and IMJM, but not SCC, compared to manual (Fig. 2B).

Next, we calculated the difference in the number of cFos-IR cells counted between manual and each automated method. An ANOVA of the total absolute errors (ABS) per method revealed a significant effect (F_2, 141_ = 30.41, *p* < 0.0001). Post-hoc analysis revealed that the ABS between manual and automated counts by SCC was significantly less than both OCFU and IMJM (Bonferroni’s test, *p* < 0.01). Therefore, compared to IMJM and OCFU, SCC had the least difference in cell counts compared to manual analysis (Fig. 2C).

**Figure 3:**
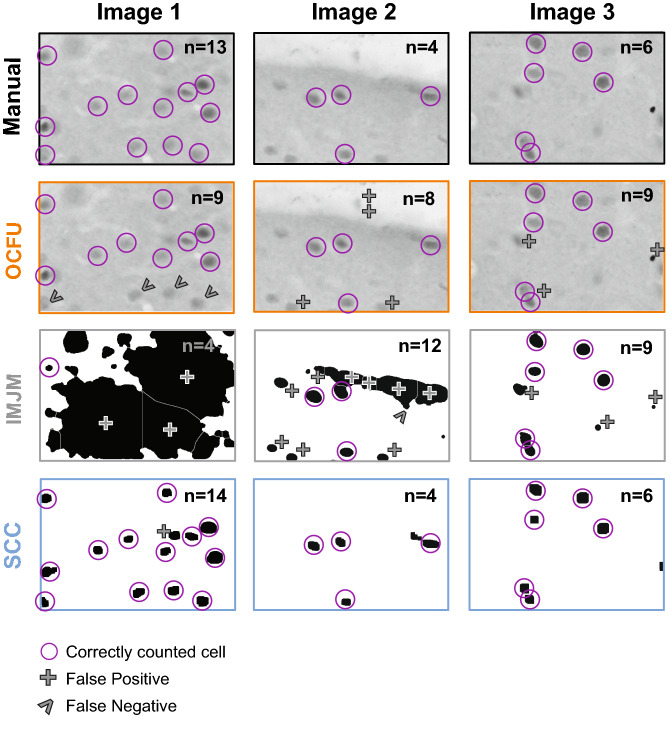
Characterization of Automated Quantification. Comparison of three images with manual versus automated method with examples of count classification: OpenColonyFormingUnit (OCFU, orange), ImageJ Edge Detection Macro (IMJM, gray) and SimpylCellCounter (SCC, blue). The number of detected cells (n) is displayed in the upper right corner of each image. Correctly counted cells (compared to manual) are depicted in magenta circles. False positives are depicted by a plus symbol while false negatives are depicted by the carrot symbol.

To further explore the types of errors observed with each automated method, we conducted an analysis of false positives (Fig. 2D) and false negatives (Fig. 2E). An ANOVA of false positives revealed a significant effect (F_2, 87_ = 22.37, *p* < 0.0001) with SCC displaying a significantly lower magnitude of false positives compared to OCFU and IMJM (Bonferroni’s test, *p* < 0.01). Additionally, an ANOVA of false negatives revealed a significant effect (F_2,87_ = 5.27, *p* < 0.01) with SCC detecting a significantly lower magnitude of false negatives compared to IMJM (Bonferroni’s test, *p* < 0.01) but not OCFU. Therefore, SCC minimized detection of false positives and negatives, resulting in a smaller ABS compared to OCFU and IMJM. Examples of types of false positives (plus symbol) and negatives (carrot symbol) compared to manual counts (magenta circles) are depicted for each automated method. The total number of cells counted (*n*) in the representative images are noted in the upper right-hand corner (Fig. 3).

Lastly, the number of cFos-IR cells quantified with SCC was correlated with manual counts (Fig. 2F). Linear regression of manual versus automated counts revealed the following: manual versus OCFU, *p* < 0.0001 with a regression equation of y = 0.552x + 20.75; manual versus IMJM, *p* < 0.0001 with a regression equation of y = 0.540x + 28.68; manual versus SCC, *p* < 0.0001 with a regression equation of y = 0.948x + 0.31. Therefore, SCC detected similar cell counts per image when compared to manual analysis.

### Differences in automated method performance (Experiment 2)

**Figure 4:**
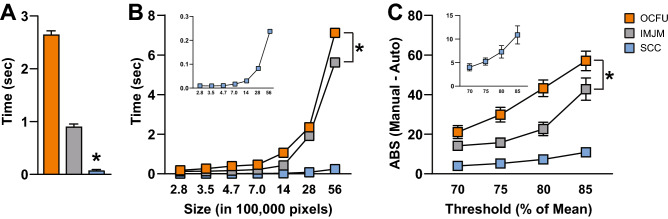
Performance Analytics of Automated Methods. OpenColonyFormingUnit (OCFU, orange), ImageJ Edge Detection Macro (IMJM, gray) and SimpylCellCounter (SCC, blue) performance analytics were compared. (**A**) Average time (sec) to quantify cFos-Immunoreactive (IR) cells per image (1920 × 1460 pixels), n = 30 images per automated method. (**B**) Average time (sec) as a function of image size (pixels) to quantify the number of cFos-IR cells per image, n = 30 images per automated method. Inset displays data for SCC only. (**C**) Average absolute error per image where: $ABS\left(Manual\;counts-Automated\;counts\right)$ as the binary threshold value approaches the mean pixel intensity of the image, n = 15 images per threshold factor. Inset displays data for SCC only.

We compared the performance analytics of OCFU, IMJM and SCC at analyzing cFos-IR cells (Fig. 4). We determined the average time (sec) for each automated method to quantify one image (Fig. 4A). An ANOVA of time per method revealed a significant effect (F_2, 87_ = 1,292, *p* < 0.0001) with SCC exhibiting the fastest analysis time (Bonferroni’s test, *p* < 0.01). The time required to quantify a set of images as a function of image size was also compared (Fig. 4B). A 3 × 7 repeated measures ANOVA of analysis time per method revealed a significant Method x Size interaction effect (F_12, 522_ = 2,271, *p* < 0.0001) and a significant main effect of Size (F_6, 522_ = 9,773, *p* < 0.0001) with SCC exhibiting the fastest time to analyze one image across size groups (Bonferroni’s test, *p* < 0.01). Therefore, all three automated methods display increases in processing time with larger image size. While SCC’s processing time increases as a function of image size, it exhibits the most rapid analysis compared to OCFU and IMJM.

Lastly, we compared the ABS of each automated method as the threshold varied as a percentage of mean pixel intensity per image (Fig. 4C). A 3 × 4 repeated measures ANOVA of ABS per method revealed a significant Method x Threshold group interaction effect (F_6, 126_ = 9.11, *p* < 0.0001) and a significant main effect of Threshold group (F_3, 126_ = 66.08, *p* < 0.0001) with SCC displaying the lowest ABS across Threshold groups (Bonferroni’s test, *p* < 0.01). Therefore, SCC displays robust accuracy even as threshold percentage and background noise increases.

### Determining SCC’s performance on different image types (Experiment 3)

**Figure 5:**
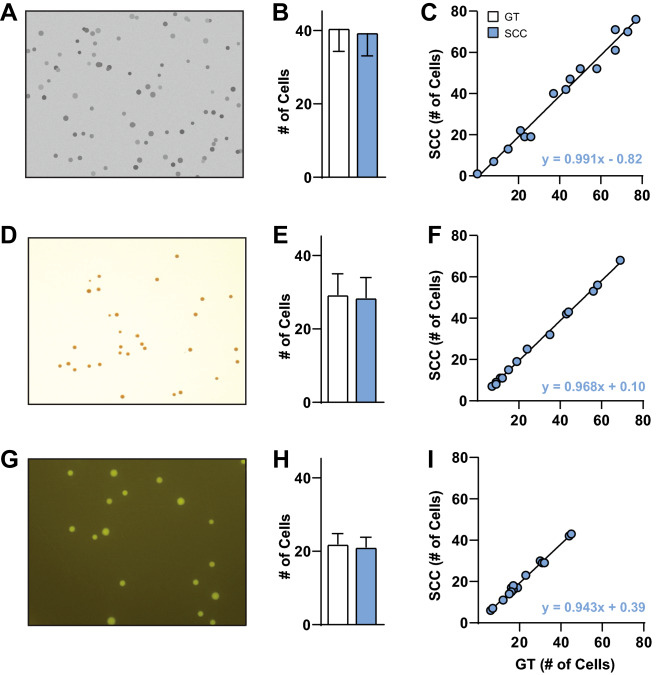
Overfitting Analysis. SimpylCellCounter (SCC) evaluated multiple sets of data including fabricated cFos images and non-neuronal cell types *S. aureus* and *E. coli* to test the generalizability of our algorithm. Ground truth (GT) was defined as: the number of known cFos-Immunoreactive (IR) cells that meet threshold, size, and circularity criterion on fabricated cFos images (**A**–**C**) and the number of cells per image counted by OpenColonyFormingUnit (OCFU) (**D**–**I**). OCFU was trained on these two datasets, thereby making it GT. GT (white bars), SCC (blue bars). (**A**) Representative image of fabricated cFos cells, (**B**) Average number of cells counted by GT versus SCC, n = 15 images, (**C**) GT counts correlated with SCC counts resulted in *p* < 0.001 and regression equation of y = 0.991x − 0.82, (**D**) Representative image of *S. aureus*, (**E**) Average number of GT cells versus SCC, n = 14 images, (**F**) GT counts correlated with SCC counts resulted in *p* < 0.001 and regression equation of y = 0.968x + 0.10, (**G**) Representative image of *E. coli*, (**H**) Average number of GT cells versus SCC counts, n = 15 images, (**I**) GT counts correlated with SCC counts resulted in, *p* < 0.001 and regression equation of y = 0.943x + 0.39.

We compared ground truth (GT, white) to SCC (blue) counts on three different types of images: fabricated cFos-images, *S. aureus* and *E. coli* cell colonies (Fig. 5). Independent samples t-test of average GT fabricated cFos-image counts versus SCC counts revealed no significant differences (Fig. 5B, t_28_ = 0.13, *p* = 0.89). Linear regression of GT fabricated cFos-image counts versus SCC counts revealed a significant correlation (Fig. 5C, *p* < 0.0001) and regression equation of y = 0.991x − 0.82. Independent samples t-test of average GT *S. aureus* counts versus SCC counts revealed no significant differences between groups (Fig. 5E, t_26_ = 0.11, *p* = 0.91). Linear regression of GT *S. aureus counts* versus SCC counts revealed a significant correlation (Fig. 5F, *p* < 0.0001) and regression equation of y = 0.968x + 0.10. Independent samples t-test of average GT *E. coli* counts versus SCC counts revealed no significant differences between groups (Fig. 5H, t_28_ = 0.20, *p* = 0.84). Linear regression of GT *E. coli* counts versus SCC counts revealed a significant correlation (Fig. 5I, *p* < 0.0001) and regression equation of y = 0.943x + 0.39. Taken together, these results show that SCC counts matched GT counts for fabricated cFos images (Fig. 5A–C), *S. aureus* images (Fig. 5D–F) and *E. coli* colonies (Fig. 5G–I). Therefore, SCC accurately quantifies cell types that it was not trained on, indicating generalizability.

## Discussion

The present study aimed to create an automated method to analyze cell number in brain tissue to complement existing open-sourced methods designed for cell colony analysis. We created SimpylCellCounter (SCC) and used this automated method to quantify the number of cFos-immunoreactive (cFos-IR) cells in brain tissue. We analyzed several variables and found that SCC (1) detected a similar magnitude of cells as manual analysis, (2) displayed low absolute errors (ABS), false positives and negatives compared to two widely-used automated methods OpenColonyFormingUnit (OCFU) and ImageJ Edge Detection Macro (IMJM), (3) was rapid at processing images of increasing size and (4) detected similar number of cells across varying thresholds suggesting that this algorithm can maintain accuracy when background noise changes. Importantly, SCC introduces a novel approach to detect and quantify overlapping cells with the use of Hu Moments and a convolutional neural network (CNN). Use of a pretrained CNN affords rapid analysis along with high levels of accuracy at quantifying overlapping cells. For binarized objects with significant overlap, it is difficult to judge whether there are multiple cells or simply one cell with irregular morphological features. Our CNN implementation is optimal for such a task since it learns morphological features of binarized single or multiple cells, thereby making input data classification with a pretrained network a quick process.

SCC-driven analysis of cFos-IR cell number was correlated with manual counts. We previously found that rats presented with a drug-associated cue displayed an increased number of cFos-IR cells in the ventral orbitofrontal cortex (vOFC). When compared to previous manually-analyzed data, SCC detected similar numbers of total cFos-IR cells (Fig. 2B**)** and similar numbers of counts in anterior–posterior divisions of the OFC (Fig. 2A). There was a near perfect match of cell counts analyzed manually versus with SCC (Fig. 2F). Taken together, these data indicate that SCC is comparable to manual analysis, accurate at providing objective estimates of cell number and efficient since less time is needed to determine whether a cell should be counted based on size, shape and pixel intensity.

We compared manual analysis of cell counts to two commonly-used algorithms OCFU and IMJM which analyze cell number in colonies but can also be used for other applications requiring detection of circular objects. The method by which OCFU and IMJM filter out noise particles from images is distinct. OCFU extracts features of an object on an image by determining whether they are part of the foreground or background, whereas IMJM uses edge detection followed by multiple filtering steps. SCC was not benchmarked against general cell analysis methods like CellProfiler^20^ and Ilastik^21^ since they require the user to have a working understanding of computer vision algorithms to create a custom pipeline for analysis prior to inputting data, making these solutions less user-friendly. In addition, we did not compare SCC to more sophisticated deep-learning approaches such as Deep FLASH. For users with computational proficiency and access to high-level computing hardware, this algorithm would be a useful resource^22^.

We also examined whether OCFU, IMJM and SCC detected similar numbers of cells. While not significant, there was a trend for OCFU and IMJM to result in higher total numbers of cFos-IR cells in the vOFC and across bregmas compared to manual and SCC (Fig. 2B). When we compared the ABS between manual and automated counts, we found that SCC resulted in significantly less errors than OCFU and IMJM (Fig. 2C). We then aimed to understand the potential reasons for differences in cell number between manual, OCFU, IMJM and SCC methods. Examples of false negatives occurred where OCFU filtered out cFos-IR cells that were oblong shaped or blurry. False positives may have occurred when cells displayed a color gradient (half of cell dark, other half light), resulting in a cell being counted twice (Fig. 3, OCFU). Examples of false negatives in IMJM occurred when contrast enhancement created a loss of difference in pixel intensity between a cell versus background, resulting in edge detection failure and omission of neighboring positive cells. Additionally, noise filtering procedures may alter cell morphology, making it difficult for IMJM’s watershed algorithm to effectively separate certain overlapping cells, leading to lower counts (Fig. 3, IMJM). False positives could result from errors where background, noise-like particles with correct cell shape are erroneously filled with IMJM’s “fill holes” step, leading to increased counts.

SCC is a brain-specific algorithm that complements currently-available, automated quantification methods, offering improvements in speed of digital analysis. SCC’s simple processing scheme only includes functions that are essential to separating cFos-IR cells from background and noise, such as thresholding, dilation and erosion. Similar to OCFU and IMJM, SCC initially processes an entire digital image until step 4 of the algorithm (Fig. 1A). Then SCC computes Hu Moments to quantify circularity independently and sequentially in a contour-wise fashion. Therefore, SCC decides which objects demand additional time for analysis: non-circular contours are input into the CNN to test for overlapping cells while circular objects are simply counted thereby reducing processing times (Fig. 4A). We also determined that SCC is consistently faster at processing increasingly larger images (Fig. 4B) and maintains a high level of count accuracy even as threshold values change (Fig. 4C). As thresholds approach the mean pixel intensity of the image being processed, the ABS between manual and automated counts consistently increases for all automated methods. However, SCC minimizes this error likely due to the dynamic filtering operations: as threshold approaches the mean, objects are more rigorously filtered by increasing iterations of dilation and erosion.

Lastly, we aimed to determine whether the SCC algorithm could also accurately analyze cell counts from other lab data. To do this, we utilized data from artificially-constructed cFos-IR images that contained cells varying in size and intensity. SCC exhibits accurate performance as shown by raw averages and correlation (Fig. 5A–C). Furthermore, we utilized sample images obtained from the OCFU database from *S. aureus* and *E. coli* image samples. SCC displayed strong correlations between ground truth versus automated detection method, demonstrating that our algorithm can detect circular, non-cFos-IR cells that are pigmented and from in vitro mediums (Fig. 5D–F, G–I). These data demonstrate that SCC is not overfit to the data it was trained on and can likely generalize to other datasets collected by different labs. While SCC can count other types of cells in bacterial cultures, it is built for cFos-like images.

In the future, SCC can potentially be used to effectively analyze viral or fluorescent images. For example, colorimetric cFos images have dark cells and a lighter background, whereas fluorescently-labeled cells would be lighter than the background. Therefore, the user can simply invert the threshold in SCC and proceed with the same processing chain. Given that the SCC code is flexible, a user can easily adjust parameters of threshold, size and filtering to fit their specific application. SCC is an accurate, efficient and novel automated tool to quantify cFos-like cells in brain tissue and can be extended to analysis of cells with circular morphologies.

## Materials and methods

### Animals and behavioral experiments

We utilized a previously published data set in which the number of cFos-IR cells was increased with exposure to cocaine-associated cues^23^. Male Sprague–Dawley rats (Envigo Inc, Haslett, MI, N = 20) were housed under reversed lighting conditions (lights off 7 am, on 7 pm) and were fed 20–25 g of standard irradiated rodent chow with water available ad libitum. Protocols were approved by the Institutional Animal Care and Use Committee (IACUC) at Michigan State University (MSU) and followed the National Research Council’s Guide for the Care and Use of Laboratory Rats. Intravenous catheters were subcutaneously implanted into the right jugular vein. Following 5 days of recovery, rats underwent cocaine self-administration and extinction training followed by drug-seeking tests without cue (EXT) or with cue presentation (TEST). After tests, rats were sacrificed and perfused, brains were extracted and cryoprotected and sectioning and image processing was conducted as previously described^23^. We obtained a total of 192 images of cFos-stained sections from lateral and ventral OFC (lOFC, vOFC respectively) from six bregma points spanning the anterior-posterior axis (+ 5.12 to + 3.72), from which cFos-IR cells were quantified^23^. In the current study, we compared counts that were previously analyzed manually with the automated methods OCFU, IMJM and SCC.

### Image processing steps and parameter selection

#### Manual

We imported images into ImageJ*v*1.51^13^ and converted them to 8-bit grayscale. Threshold values of 115 (top value) and 120 (bottom value) were applied so as to selectively quantify darkly-stained cFos-IR cells^23^. These adjustments created a round, red contour around the darkest cells on each image which assisted experimenters to judge the circularity and size of cells during counting (Fig. 1A). For all automated methods (OCFU, IMJM and SCC) parameters such as radius size, contrast enhancement and circularity values were set and adjusted to correspond with our manual value settings.

#### OCFU

We utilized the OCFU graphic user interface application for all experiments. We set two parameters: radius size (minimum at 10 pixels, maximum at auto-max) and threshold to 61. Since OCFU’s threshold does not directly correspond to pixel intensity, we incorporated a standardization procedure (Supplementary Methods and Supplementary Fig. S3) by which a threshold value of 61 in OCFU was equivalent to the value of 115 in manual, IMJM and SCC. (Fig. 1A).

#### IMJM

This algorithm contained numerous parameters that were local to ImageJ functions but not common to OCFU or SCC. Therefore, we used parameters for IMJM provided previously^16^ but modified the contrast enhancement value to 0.001 and circularity value to 0.8–1.0 (Fig. 1A). We also added a binary threshold step and implemented IMJM in MATLAB’s ImageJ wrapper, MIJI^24^. The exact MIJI workflow can be found at: https://github.com/aneeshbal/SimpylCellCounter/blob/master/recreationFunctions/AutoQMS_MIJI.m

#### SCC

Binary Mask: In the first processing step, the user selects the folder of images to be analyzed and then SCC applies a binary threshold to 8-bit images, converting all pixel values lower than the set threshold to black and those higher than threshold to white. This binary threshold value is set by the user and can be changed to any pixel intensity value (0–255). The values were adjusted to detect dark-stained, rather than light-stained, cFos-IR cells. After thresholding, shapeless, poorly-connected, sparse groups of black pixels represent background noise-like particles while round, well-connected, dense collections of black pixels represent cFos-IR cells.

Dilation and Erosion: In the dilation step, white pixels (background) engulf adjacent black pixels (cells of interest + noise). Consequently, small, noise-like objects are completely engulfed by white pixels and become part of the background. To recover an object’s original morphology, which is altered with dilation, SCC performs an erosion step (opposite of dilation). Dilation and erosion steps occur for a set number of iterations, determined by the user-set threshold to mean pixel intensity ratio (MPI). As the threshold-MPI ratio approaches 1, the magnitude of noise-like particles exponentially increases. Therefore, SCC accordingly increases the iterations of these steps resulting in a stringent filtering process.

Object selection: Following dilation and erosion, SCC discards objects based on size criteria by drawing contours over all the objects on the filtered binary mask, calculates the zeroth-order moment of each contour (area) and discards all contours with a smaller area than the user-set criteria (pixel radius converted to area). Following this step, certain cells that are overlapping, may obscure the total cell count. Rather than performing the popular watershed segmentation algorithm to separate overlapping cells, which can alter cell morphology^25^, we utilized Hu Moments to compute contour circularity^26,27^. Hu Moments are orientation- and scale-invariant properties intrinsic to shapes. Perfectly circular contours resulted in a log-adjusted first Hu Moment value of ~ 0.79. We observed that a single contour surrounds the perimeter of overlapping cells, resulting in a non-circular contour with a first Hu Moment value typically below ~ 0.76. SCC then applies a pretrained convolutional neural network (CNN) classifier (Supplementary Methods and Supplementary Fig. S1) to determine the number of cells within non-circular contours and adds these to the number of circular contours. For all experiments, radius size was set to 10 pixels, threshold was 115 and circularity was 0.7 unless otherwise stated. (Fig. 1A). We also show that CNN is necessary for accurate counts, effective at separating overlapping cells and that SCC processing time is dependent on the number of overlapping cells in an image (Supplementary Fig S2D, E).

### Experiment 1: accuracy and feasibility of automated methods

Using 192 images of vOFC brain sections from TEST rats, we compared the average number of cFos-IR cells per bregma point and the total number of cFos-IR cells. We calculated the ABS of cell counts between manual versus each automated method (OCFU, IMJM, SCC) and then conducted an error analysis in a subset of images. For each manually-counted cell, the number of false positives (cells counted by automated methods but not manually) and false negatives (cells counted manually but not by automated methods) were determined. Last, we correlated the number of counts detected via manual versus each automated method and conducted a linear regression analysis.

### Experiment 2: performance analytics of automated methods

Using 30 randomly selected cFos-IR images from EXT and TEST subjects (lOFC and vOFC), we calculated the average time (sec) to process one image (1920 × 1460 pixels), then resized each image (by factors of 0.5, 1, 2, 4, 6, 8, 10) and calculated the resulting image size: $New \; Image\;  Size=\frac{Original\;  Image Size}{Resize\;  Factor}$. For each resize factor, we calculated the average time (sec) for each automated method to process 30 images. Lastly, using 15 randomly-selected cFos-IR images from EXT and TEST subjects (lOFC and vOFC), we calculated the ABS for each automated method as a function of a changing threshold. For each image, we multiplied the MPI by each threshold factor (0.7, 0.75, 0.8 or 0.85) to obtain the final threshold value. We then quantified cFos-IR cells and the ABS for each image between manual versus automated methods. We did not use the command line interface for OCFU but instead quantified the image processing time on the graphic user interface from the input image until a cell count was displayed.

### Experiment 3: overfitting analysis for SCC

We obtained three separate datasets of non-cFos images including: (1) fabricated cFos images (n = 15), (2) *S. aureus* colony images (n = 14) and (3) *E. coli* colony images (n = 15). We created 15 fabricated cFos images using a Python implementation of OpenCV by placing a random number of circles (between 5 and 100) of varying pixel intensities and sizes on a gray background that closely resembles the background staining of cFos images. Additionally, we obtained cell colony images of *S. aureus* and *E. coli* from the open-sourced database provided by Dr. Quentin Geissman at the following link: http://opencfu.sourceforge.net/samples.php. The source images contain agar plates but since SCC does not have a region-of-interest selector, images were cropped to include only a subset of contents inside agar plates.

For fabricated cFos-IR images, we calculated GT counts and compared them to SCC counts. GT here was defined as the number of cells that met the user-defined, size and threshold criteria. Since these images were fabricated, the exact number of cells was pre-determined. We calculated the average counts per image and performed a linear regression of GT versus SCC counts. For cell colony images, we defined GT as the number of cells counted by OCFU given that it was optimized for cell colony images. We then repeated the analyses performed on the fabricated cFos-IR images on *S. aureus* and *E. coli* image samples.

### Statistics

For Experiments 1 and 2, repeated measures analysis of variance (ANOVA) were conducted for mean cell counts across bregma points comparing manual versus automated methods (within-subject factor = bregma, between-subject factor = method), time per automated method across image size groups (within-subject = image size, between-subject = method) and ABS by automated methods across threshold factor (within-subject = threshold group, between-subject = method). One-way ANOVAs were conducted to explore differences in total cell counts, ABS, false positives and negatives across automated methods and time taken to analyze images per automated method. Additionally, linear regression was conducted to examine correlations between manual versus automated counts and GT versus SCC counts. For Experiment 3, independent t-tests were conducted to examine differences between GT versus SCC counts. For all statistical and post-hoc tests, alpha was set to 0.05.

## Preprint

A previous version of this manuscript was published as a preprint on bioRxiv [28].

## Supplementary information

Supplementary file1 (DOCX 35 kb)

Supplementary file2 (EPS 1495 kb)

Supplementary file3 (EPS 1703 kb)

Supplementary file4 (EPS 955 kb)

## Data Availability

All code is available: https://github.com/aneeshbal/SimpylCellCounter.
