# Supplementary material for: SimpylCellCounter: an automated solution for quantifying cells in brain tissue
Source: Sci Rep. 2020 Jul 28;10:12570. doi: 10.1038/s41598-020-68138-4 (PMC7387348; doi:10.1038/s41598-020-68138-4)
Supplement: Supplementary file 1 — Supplementary file1 (DOCX 35 kb) [file 41598_2020_68138_MOESM1_ESM.docx]

**SimpylCellCounter: An Automated Solution for Quantifying Cells in Brain Tissue**

Aneesh Bal^1^, Fidel Maureira^2^, Amy A. Arguello^1*^

^1^Psychology Dept., Michigan State University,

Interdisciplinary Science and Technology Building, West Lab Rm 4100

766 Service Rd., East Lansing, MI, 48824, USA

^2^Biological Systems Engineering, Washington State University, Paccar 351

Pullman, WA, 99164-6120, USA

*Corresponding Author:

Dr. Amy A. Arguello

Michigan State University

Department of Psychology, Behavioral Neuroscience

Interdisciplinary Science and Technology Building, West Lab Rm 4100

766 Service Rd

East Lansing, MI 48824

Phone: 214-912-8911

Email: [arguell5@msu.edu](mailto:arguell5@msu.edu)

**SUPPLEMENTARY METHODS**

***Convolutional Neural Network (CNN) Description***

We formulated a novel approach to detecting overlapping cells in any given non-circular contour by utilizing a convolutional neural network (CNN) classifier (Supplementary **Fig S1**). Our CNN consisted of an 8-layer network built in a Python implementation of a Keras Sequential model^1–3^. The first layer is a convolutional input layer followed by 3 down-sampling layers and 2 up-sampling layers, inspirited by the U-net architecture^4^. Following the convolutional layers, the output is flattened and is inputted into a 32-neuron, fully-connected dense layer. Lastly, this 32-neuron layer fully connects to a 3-neuron, dense layer which makes the final classification for number of cells^5^.

The network was trained to classify either 1, 2 or 3 cells in any individual contour. The training and validation data consisted of 30,000 fabricated images of cells, each image containing either 1, 2 or 3 cells on a 100 x 100 pixel square. By doing this, the network was exposed to many examples of 2 or 3 overlapping cells and was able to appropriately classify them. The network was trained on Google’s Colab GPU services for 15 epochs (24,000 samples per epoch), achieving a training accuracy of 96.10% and a validation accuracy of 90.50%. It should be noted that a sub-95% validation accuracy is to be expected on such a task given that if two cells were perfectly overlapping, it would be nearly impossible to classify the image accurately. Though the CNN is pre-trained and highly-optimized for SCC, the code for retraining the CNN or modifying its architecture is available at: <https://github.com/aneeshbal/SimpylCellCounter/blob/master/recreationFunctions/CNN_for_Overlap.ipynb>

***Effect of CNN on (SimpylCellCounter) SCC Image Analysis***

SimpylCellCounter’s (SCC) CNN module is only applied to non-circular contours that likely contain overlapping cells. Generally, we observed very few examples in which more than three cFos cells were overlapping. However, to test whether CNN improves the accuracy of cell counts with increasing number of overlapping cells, we conducted an experiment with 100 cFos-like images, in which we added 50 to 150 cells, resulting in a range of 1 to 20 overlapping cells per image. We analyzed these modified images via SCC with and without CNN (SCC +/- CNN). For each image, the number of counts per image, overlapping cells, and ground truth (GT) counts between SCC +/- CNN was determined. We also compared the average absolute error (ABS) of GT *vs* SCC +/- CNN and correlated ABS with the number of overlapping cells per image (**Fig 2**, Supplementary **Fig S2A-C**). We also recorded the time (sec) to complete each image analysis to test whether differences were observed with SCC +/- CNN. This time analysis was also benchmarked against OpenColonyFormingUnit (OCFU) and ImageJ Edge Detection Macro (IMJM) and the number of overlapping cells per image was correlated with analysis times for OCFU, IMJM and SCC +/- CNN (Supplementary **Fig S2D-E**). All SCC +/- CNN analyses were conducted on a Google Colab notebook and utilized the GPU accelerator: <https://github.com/aneeshbal/SimpylCellCounter/blob/master/recreationFunctions/scc_cnn_time_and_accuracy.ipynb>

***OCFU Threshold Standardization***

OCFU’s definition of threshold relates to the relative “darkness” of colonies compared to the background intensity with higher threshold values, resulting in a more stringent analysis. With IMJM and SCC, the threshold value strictly indicates the pixel intensity at which a binary threshold will be applied. Therefore, we aimed to standardize OCFU threshold values to IMJM and SCC. To achieve this we created 255, 12-pixel radius circles on a white background that each varied by 1-pixel intensity (0-255). Then, we iterated through OCFU threshold values starting from 0 to 255 and determined how many cells were counted. The number of counted cells at any given OCFU threshold value represented the equivalent threshold value in IMJM and SCC. Therefore, we were able to accurately compare relative threshold values across all automated methods (Supplementary **Fig S3**).

**SUPPLEMENTARY RESULTS**

***Effects of CNN on SCC Image Analysis (Supplementary Experiment 1)***

We explored whether the accuracy of SCC-determined cell counts, with or without CNN (+/- CNN) changes with increased number of overlapping cells (Supplementary **Fig S2**). The number of GT counts was correlated between SCC +/- CNN (Supplementary **Fig S2A**). Linear regression of the GT cell counts correlated with automated SCC +/- CNN, revealed the following: GT *vs* SCC + CNN, p < 0.001 with a regression equation of y = 0.888x + 4.73; GT *vs* SCC - CNN, p < 0.001 with a regression equation of y = 0.734x + 12.06.

Next, we computed the ABS between GT counts *vs* SCC +/- CNN. An independent samples t-test revealed a significant difference between the ABS of SCC + CNN *vs* SCC - CNN counts (Supplementary **Fig S2B**, t_198_ = 8.016, p < 0.001). In addition, linear regression of overlapping cells and ABS of SCC + CNN *vs* SCC - CNN revealed the following: overlapping cells *vs* ABS of SCC + CNN, p < 0.001 with a regression equation of y = 0.547x + 1.58; overlapping cells *vs* ABS of SCC - CNN, p < 0.001 with a regression equation of y = 1.534x + 0.71 (Supplementary **Fig S2C**).

Lastly, we compared the effect of CNN on the time (sec) to analyze the number of overlapping cells between OCFU, IMJM, SCC +/- CNN (Supplementary **Fig S2D**). The comparison of SCC +/- CNN is shown in **Fig S2E**. Linear regression of overlapping cells *vs* analysis time of OCFU, IMJM, SCC +/- CNN revealed the following: overlapping cells *vs* OCFU, p = 0.055 with a regression equation of y = 0.004x + 0.68; overlapping cells *vs* IMJM, p = 0.28 with a regression equation of y = 0.004x + 0.93; overlapping cells *vs* SCC + CNN, p < 0.001 with a regression equation of y = 0.0005x + 0.03; overlapping cells *vs* SCC - CNN, p = 0.105 with a regression equation of y = 0.0001x + 0.02.

Overall, the data from these experiments suggest that the addition of CNN to SCC, compared to SCC alone, resulted in increased processing time for cells counts as the number of overlapping cells increases. However, the addition of CNN also improved the accuracy of cell counts as the number of overlapping cells increases. The addition of CNN to SCC, compared to IMJM and OCFU, also resulted in faster processing times.

**SUPPLEMENTARY FIGURE LEGENDS**

**Supplementary Figure 1: Neural Network Design.** SimpylCellCounter (SCC) utilizes an 8-layer convolutional neural network (CNN) to classify overlapping cells in non-circular contours. Classification begins by SCC extracting non-circular contours following filtering in step 3 (**Fig 1B**). Non-circular contours are pasted in the center of a 100 x 100 white background, and this constitutes the input layer to the CNN. The input layer is then fed forward to a convolutional layer while down-sampling is performed via a rectified linear unit activation function (ReLU) for 4 iterations. Then, for 2 iterations, up-sampling is performed via ReLU followed by flattening. A 32-neuron, fully connected dense layer (FCDL) then receives this flattened input and feeds it forward to a 3-neuron FCDL. This final 3-neuron layer comprises the output layer which performs the final classification. ReLU activation = rectified linear unit activation function; down-sampling = reducing dimensionality of an input to allow for assumptions about its features; up-sampling = recovers the lost resolution from down-sampling, FCDL = fully-connected dense layer where all neurons connect to all input neurons, and fully connect to their feedforward layer neurons; output neuron = final neuron activation that determines classified categories.

**Supplementary Figure 2: Performance Effects of CNN on SCC Image Analysis.** Effects of the use of CNN on SCC on both accuracy of cell counts as a function of overlapping cell number. Ground truth (GT) was defined as: the number of cells that were present on the image. OCFU (orange), IMJM (gray), standard SCC + CNN (white) and SCC - CNN (blue). **A)** Correlation of GT *vs* SCC +/- CNN. GT correlated with SCC + CNN, p < 0.001, y = 0.888x + 4.73; GT correlated with SCC - CNN, p < 0.001, y = 0.734x + 12.06. **B)** Average absolute error: $ABS\left( Manual counts-Automated counts \right),$automated counts consisted of: SCC +/- CNN. **C)** Correlation of the number of overlapping cells to ABS. Overlapping cells correlated with ABS of: SCC + CNN, p < 0.001, y = 0.547x + 1.58; SCC - CNN, p < 0.001, y = 1.534x + 0.71. **D)** Correlation of the number of overlapping cells and time (sec) to analyze image. Overlapping cells correlated with: OCFU, p = 0.055, y = 0.004x + 0.68; IMJM, p = 0.28, y = 0.004x + 0.93; SCC + CNN, p < 0.001, y = 0.0005x + 0.03; SCC - CNN, p = 0.105, y = 0.0001x + 0.02. **E)** Inset of panel D), Correlation of the number of overlapping cells and time (sec) to analyze image for SCC +/- CNN. Overlapping cells correlated with: SCC + CNN, p < 0.001, y = 0.0005x + 0.03; SCC - CNN, p > 0.05, y = 0.0001x + 0.02. Symbols denote *p<0.05.

**Supplementary Figure 3: OCFU Threshold Standardization.** Standardization of OCFU’s threshold function, allows for accurate comparison to IMJM and SCC thresholds. X-axis represents the user-defined, binary pixel value threshold. Y-axis represents the corresponding threshold parameter for each respective method. For example, if the user selects 115 as a pixel value to threshold, then the OCFU threshold value to choose (orange line) is approximately 62. OCFU (orange), IMJM (gray), SCC (blue).

**REFERENCES**

1. Gu, J. *et al.* Recent Advances in Convolutional Neural Networks. *arXiv* (2015).

2. Sultana, F., Sufian, A. & Dutta, P. Advancements in Image Classification using Convolutional Neural Network. *arXiv* (2018).

3. Koushik, J. Understanding Convolutional Neural Networks. *arXiv* (2016).

4. Ronneberger, O., Fischer, P. & Brox, T. U-Net: Convolutional Networks for Biomedical Image Segmentation. *arXiv* (2015).

5. Huang, G., Liu, Z. & van der Maaten, L. Densely Connected Convolutional Networks. *arXiv* (2018).
